# Factors correlating the expression of PD-L1

**DOI:** 10.1186/s12885-024-12400-9

**Published:** 2024-05-25

**Authors:** Fang Lu, Ernuo Wang, Haiquan Liu

**Affiliations:** Department of Radiology, Huadong Hospital, Fudan University, Shanghai, 200040 China

**Keywords:** PD-L1, Ki67, EGFR, Smoking, pGGO, Lung adenocarcinoma, The high-resolution computed tomography (HRCT)

## Abstract

**Objective:**

PD-L1 was an important biomarker in lung adenocarcinoma. The study was to confirm the most important factor affecting the expression of PD-L1 remains undetermined.

**Methods:**

The clinical records of 1045 lung adenocarcinoma patients were retrospectively reviewed. The High-Resolution Computed Tomography (HRCT) scanning images of all the participants were analyzed, and based on the CT characteristics, the adenocarcinomas were categorized according to CT textures. Furthermore, PD-L1 expression and Ki67 index were detected by immunohistochemistry. All patients underwent EGFR mutation detection.

**Results:**

Multivariate logistic regression analysis revealed that smoking (OR: 1.73, 95% CI: 1.04–2.89, *p* = 0.004), EGFR wild (OR: 1.52, 95% CI: 1.11–2.07, *p* = 0.009), micropapillary subtypes (OR: 2.05, 95% CI: 1.46–2.89, *p* < 0.0001), and high expression of Ki67 (OR: 2.02, 95% CI: 1.44–2.82, *p* < 0.0001) were independent factors which influence PD-L1 expression. In univariate analysis, tumor size > 3 cm and CT textures of pSD showed a correlation with high expression of PD-L1. Further analysis revealed that smoking, micropapillary subtype, and EGFR wild type were also associated with high Ki67 expression. Moreover, high Ki67 expression was observed more frequently in tumors of size > 3 cm than in tumors with ≤ 3 cm size as well as in CT texture of pSD than lesions with GGO components. In addition, multivariate logistic regression analysis revealed that only lesions with micropapillary components correlated with pSD (OR: 3.96, 95% CI: 2.52–5.37, *p* < 0.0001).

**Conclusion:**

This study revealed that in lung adenocarcinoma high Ki67 expression significantly influenced PD-L1 expression, an important biomarker for immune checkpoint treatment.

## Background

Lung cancer is the major cause of increased mortality by cancer worldwide, and Non-small cell lung cancer (NSCLC) accounts for approximately 85% of all lung cancer cases. Lung adenocarcinoma (LUAD) is the most common pathological subtype of NSCLC [1].

Computed Tomography (CT) is used for diagnosing lung cancer, and persistent pulmonary ground-glass opacity (GGO) on CT is closely associated with LUAD [2]. In high-resolution CT (HRCT), GGO is defined as hazy pulmonary nodules with lesions that lack obscure bronchial structures or pulmonary vessels [3]. According to the proportion of ground glass components, pulmonary nodules can be classified as pure GGO nodules (pGGO), mixed GGO nodules (mGGO), and solid nodules (SN) [4]. In LUAD, various GGO types are associated with different prognoses [5].

Proliferation is a crucial characteristic of LUAD progression. The Ki-67 labeling index is a widely used prognostic biomarker that is estimated by the immunohistochemical labeling of nuclear antigen Ki-67 [6]. Ki67 is a DNA-binding nuclear protein, which is expressed throughout the proliferative phases of the cell cycle and absent in the quiescent (G0) phases [7, 8]. Furthermore, the literature suggests that Ki67 is correlated with the prognosis of lung cancer [9, 10].

The epidermal growth factor receptor (EGFR) is present on the cell surface and is a member of the receptor tyrosine kinase (RTK) superfamily. It mediates cell signaling by extra-cellular growth factor; therefore, targeted therapies for EGFR mutation with better clinical efficacy have been a hotspot in research [11].

In recent years, monoclonal antibodies against immune checkpoints have made breakthroughs across many tumor types, especially in melanoma, as well as lung, kidney, and bladder cancers [12]. It has been suggested that the activation of the PD-1/PD-L1 pathway helps tumor cells escape T-cell cytolysis and facilitate cancer initiation. Immune checkpoint inhibitors (ICIs) can reactivate T-cell activity by directly binding PD-1 or its ligand PD-L1, thereby blocking the PD-1/PD-L1 pathway and ultimately eliminating tumor cells [13].

The literature suggests that PD-L1 expression is correlated with male gender, smoking [14, 15], high Ki67 expression [16], wild-type EGFR status [13, 17, 18], and absence of GGO with CT texture of LUAD [3]. However, the dominant factors remain undetermined. Because of the importance of PD-L1, it is an essential research topic.

## Methods

### Patients

This retrospective study was approved by The Ethics Review Board of Huadong Hospital, Affiliated with Fudan University, and followed the Declaration of Helsinki. The requirement for informed consent was waived because the study was retrospective.

### Inclusion and exclusion criteria

According to the inclusion criteria, patients who (a) underwent surgical resection, (b) were pathologically diagnosed with LUAD, and (c) had a CT scan before surgery were included in the study. Of 2000 patients, 955 were excluded as (a) they lacked preoperative CT images (*n* = 50), (b) their lesions manifested as diffuse miliary nodules in the two lungs (*n* = 22), (c) they had multiple lesions (*n* = 335), (d) their lesions were obscured with atelectasis, pneumonia or massive pleural effusion (*n* = 31), or (e) their PD-L1 expression status was not available (*n* = 517). Finally, 1045 patients were included in this study.

### Patients’ general characteristics

All the patient’s medical records were reviewed and their data on sex, age, smoking history, Ki67, T staging, PD-L1 expression, EGFR mutation status, and pathological subtypes, were acquired. These factors were selected because (1) Ki67 is used to estimate a cell’s proliferation rate [19]. Furthermore, it has been reported that signaling pathways of PD-L1 and Ki67 interact with each other. (2) EGFR is a druggable target in LUAD [20]. (3) Smoking is a risk factor for lung cancer [21]. (4) T staging is a basic characteristic of cancer [22]. (5) The expression of PD-L1 has been associated with some subtypes [23].(6) A study showed that PD-L1 expression was associated with absence of surrounding ground glass opacity [13].

### Computed tomographic assessment

Preoperative chest CT was performed using three scanners: GE Discovery CT750 HD, 64-slice Light Speed VCT (GE Medical Systems), and Somatom Definition flash with the following parameters: 120 kVp, 100–200 mAs, pitch = 0.75–1.5, and collimation = 1–1.25 mm, respectively. All imaging data were reconstructed using a medium sharp reconstruction algorithm of 1–1.25 mm thickness.

### Interpretation of computed tomographic images

The CT images were retrospectively interpreted by a clinical radiologist (H.Q.L) with 35 years of experience and two other radiologists (F.L and E.N.W) with 10 years of experience, independently. The radiologists were blinded to clinical data. For the final CT feature value, the majority class was used, and CT images were read at both mediastinal (width = 350 HU; level = 40 HU) and lung (width = 1500 HU; level = -650 HU) window settings. Furthermore, the lesion location, size, and texture were retrospectively evaluated, and the long axis diameter at the lesion’s maximal section was measured. The lesion with only GGO was classified as pGGO, that with > 50% but < 100% was classified as a GGO predominant nodule (mGGO), while that with > 1% but ≤ 50% GGO was defined as a solid predominant nodule (mSD). Moreover, the lesions that lacked GGO were classified as a pure solid nodule (pSD). During statistical analysis, the mGGO and mSD were combined and called mGGO.

### Histopathologic analysis

The LUAD tumors were identified and classified based on the 2011 IASLC/ATS/ERS classification system [24]. Furthermore, each lesion’s histological subtype (acinar, lepidic, papillary, micropapillary, and solid) was also assessed.

The hot-spot area was determined under the low-power field for Ki67 and PD-L1 assessment. A total of 1000 tumor cells were counted under 400 × magnification, and the positive tumor cell percentage was assessed.

The samples were stained with Ki-67 antibodies for immunohistochemistry, and the positively stained cells with the highest density were quantified. A total of 1000 tumor cells were counted under 400 × magnification. The positive percentage of tumor cells was denoted as the Ki-67 labeling index. The expression values of < 10% indicate low Ki67 expression, while the expression values of ≥ 10% depict high Ki67 expression.

The immunohistochemistry of PD-L1 antibody labeled slides was performed on the Dako Autostainer Link 48 platform using the PD-L1 22C3 antibody. The tumor proportion score (TPS) was used to evaluate PD-L1 expression in tumor cells. PD-L1 positivity was evaluated using a tumor cell expression (TC) method = (number of PD-L1 stained tumor cell/total tumor cell × 100) Where, ≤ 1% = negative, > 1% = positive. All the samples were evaluated by two expert pathologists by following the method of Kim T et al. [25].

Furthermore, using histological specimens of LUAD patients, the EGFR mutation status of exon 18–21 was detected with a polymerase chain reaction-based amplified refectory mutation system (ARMS) with the help of a Human EGFR Gene Mutation Fluorescence Polymerase Chain Reaction Diagnostic Kit (AmoyDx, China).

### Statistical analysis

All the statistical analysis was performed *via* SAS 9.4 (SAS Institute Inc., Cary, NC). The differences in categorical variables distribution were evaluated by the χ2 test, and differences with *p* < 0.05 were considered statistically significant. Multiple logistic regression analyses were performed to identify independent factors that can influence the expression of PD-L1. The final model was selected with the backward elimination method, with a cutoff *p-value of 0.05*.

## Results

### The demographics

A total of 1045 patients were included in this study, including 443 men and 577 women. The age ranged from 22 to 90 years, with an average of 63.3 years old, and among these, 85 had a smoking history.

### The correlation between clinical features and expression of PD-L1

Table 1 indicates that the PD-L1 expression rates were significantly higher (a) in men [143 of 457 (31.3%)] than in women [131 of 588 (22.3%)] (OR: 1.59, 95% CI: 1.20–2.01, *p* = 0.001) (b) in smokers [38 of 85 (44.7%)] than in nonsmokers [236 of 960 (24.6%)] (OR: 2.480, 95% CI: 1.58–3.90, *p* < 0.0001) (c) in high expression of Ki67 patients [174 of 461 (37.7%)] than in low expression of KI67 patients [100 of 584 (17.1%)] (OR: 2.93, 95% CI: 2.20–3.91, *p* < 0.0001) and (d) in EGFR wild type [115 of 334 (34.4%)] than in EGFR mutation type [159 of 711 (22.4%)] (OR: 1.82, 95% CI: 1.37–2.43, *p* < 0.0001). (e) in lesions with micropapillary components [122 of 277 (44.0%)] than in without micropapillary components [153 of 768 (19.9%)] (OR: 3.2, 95% CI: 2.40–4.30, *p* < 0.0001). Although the *p-value* was *< 0.05* for the lepidic, papillary, and solid components, these pathological subtypes were negatively correlated with the expression of PD-L1. No significant association was observed between age and PD-L1 expression.

### The correlation between CT texture and expression of PD-L1

High expression of PD-L1 was more frequent in pSD [145 of 406 (35.7%)] than in pGGO [67 of 329 (20.3%)] (OR: 2.17, 95% CI: 1.55–3.04, *p* < 0.0001) (Table 1). No significant difference in PD-L1 expression was observed between pGGO and mGGO.

### The correlation between the T or N stage of the tumor and the expression of PD-L1

Tumors of ≥ T2 stage (tumor diameter > 3 cm) more frequently indicated high expression of PD-L1 [47 of 129 (36.4%)] than T1a stage [37 of 177 (20.9%)]. Furthermore, there were no PD-L1 expression differences between other stages and T1 stage tumors. Because there were reduced N-positive tumors, the N stage had no significance.

### Multivariable analysis of factors influencing the expression of PD-L1

Multivariate logistic regression analysis (of variables shown in Table 1) showed that 4 independent variables, including smoking, EGFR mutation status, Ki67 expression, and micropapillary subtype, were correlated with PD-L1 expression. High expression of PD-L1 was more frequently observed in individuals who smoked (OR: 1.73, 95% CI: 1.04–2.89, *p* = 0.004), had wild-type EGFR (OR: 1.52, 95% CI: 1.11–2.07, *p* = 0.009), high Ki67 expression (OR: 2.02, 95% CI: 1.44–2.82, *p* < 0.0001), and micropapillary subtypes (OR: 2.05, 95% CI: 1.46–2.89, *p* < 0.0001), suggesting that these were independent factors influencing PD-L1 expression (Table 2).

### Association between factors influencing the expression of PD-L1 and expression of Ki67

The association of smoking, some specific histopathological subtypes, and EGFR wild type with Ki67 expression were also analyzed (Table 3). Ki67 expression was observed more frequently in smokers [50 of 85 (58.8%)] than in nonsmokers [411 of 960 (42.8%)] (*p* = 0.004), in those with EGFR wild type [176 of 334 (52.7%)] than in those with EGFR mutation type [285 of 711 (40.1%)] (*p* = 0.0001), as well as in patients indicating lesions with micropapillary component [201 of 277 (72.6%)] than in those who did not have these lesions [261 of 768 (34.0%)] (*p* < 0.0001). There was no correlation between the solid subtype and the expression of Ki67.

### Association of tumor diameter with CT textures and Ki67 expression

The association of Ki67 expression with tumors of > 3 and ≤ 3 cm size was determined, which revealed that high Ki67 expression was more frequently observed in tumors of > 3 cm [85 of 129 (65.9%)] than in ≤ 3 cm size [376 of 916 (41.0%)] ( *p* < 0.0001). For the CT textures, Ki 67 high expression was observed more frequently in pSD tumors [278 of 406 (68.5%)] than in non-pSD tumors [183 of 639 (28.6%)] (*p* < 0.0001) (Table 4).

### Multivariable logistic regression correlation analysis between CT texture of pSD and histopathological subtypes

Multivariate logistic regression analysis (Table 5) showed that only lesions with micropapillary components correlated with pSD (OR: 3.96, 95% CI: 2.52–5.37, *p* < 0.0001).

## Discussion

Immune checkpoint inhibitors (ICIs) targeting the PD-1/PD-L1 pathway have been widely used in advance NSCLC for better efficacy and safety [26, 27]. Studies elucidated that PD-L1 ≥ 1% was positively associated with the major response (MPR), pathological complete response (PCR), 3-year overall survival (OS), and disease-free survival (DFS) [28]. Therefore, elucidating the associated factors influencing the expression of PD-L1 is essential.

This study revealed that the expression of PD-L1 was correlated with male smoking, high Ki67 expression, and wild-type EGFR in LUAD. Q. Zhu et al. also revealed an association of PD-L1 with male gender and smoking history, supported by several other studies [14, 16, 17, 29, 30]. Q. Chen et al. revealed that EGFR-mutated patients showed relatively lower PD-L1 expression than wild-type patients [17] Furthermore, it has been repetitively indicated that EGFR wild-type tumors were significantly more likely to express PD-L1 than EGFR-mutated tumors [13, 17, 18, 31–33]. M. Evans et al. analyzed PD-L1 expression in 10,005 NSCLC cases and found that EGFR wild-type tumors were significantly more likely to express PD-L1 than mutated tumors [15].

The multivariable regression analysis in this study indicated that smoking, wild-type EGFR, and high expression of Ki67 were independent factors of correlated expression of PD-L1. To elucidate the dominant factor among these three, smoking and EGFR wild type were correlated with high Ki67 expression. It was found that whether in smoker or wild EGFR patients, the frequency of high Ki67 expression was more than in counterparts. Furthermore, either in EGFR mutation or wild lesions, high expression of PD-L1 always correlated with high Ki67 expression. It is noteworthy that smoking and wild-type EGFR influenced PD-L1 expression by increasing Ki67 expression. Q. Zhu et al., by linear regression analysis, revealed a positive association between the expression of PD-L1 and Ki-67 [16]. Furthermore, Y. Zhao et al. found that patients with positive PD-L1 expression had a significantly higher incidence of more advanced tumor stage and Ki-67 index [34] Additionally, pSD is more frequently expressed in PD-L1. T. Wu et al. and K. Takada et al. demonstrated that the absence of surrounding GGO was significantly associated with the expression of PD-L1, consistent with the results of this research [13, 35]. Moreover, Mimae et al. revealed that pure solid LUAD had a more advanced T stage [36]. Studies showed that pure solid tumors exhibited more aggressive behavior (lymphatic, vascular, and pleural invasions or lymph node metastasis) [36–38]. Solid tumors exhibit more malignant behavior compared with mixed tumors [37]. Q. Chen et al. analyzed 1071 NSCLC, including 847 LUAD patients, and demonstrated that in this subgroup, high PD-L1 expression was associated with advanced T stage, consistent with other studies [17, 29, 39, 40]. Similarly, J. Zhou et al. suggested that there was almost no PD-L1 expression in AIS (adenocarcinoma in situ) or MIA (minimally invasive adenocardinoma) [41]. However, PD-L1 expression was correlated with the invasiveness of LUAD. The percentages of PD-L1 positive in IA-1, -2, and − 3 were 7.22, 11.29, and 14.20%, respectively. To compare with the study, we analyzed the PD-L1 expression in different diameters of pSD, which indicated no correlation between tumor diameter and PD-L1 expression except in those with > 3 cm size, reaching marginal statistical significance. Further analysis showed that in this group, Ki67 expression was significantly higher than its counterpart. Furthermore, Ki67 was the dominant factor influencing the expression of PD-L1. It was suggested by J. Zhou et al. [41] that reduced PD-L1 expression in AIS or MIA was due to the low expression of Ki67 and that advanced stage LUAD corresponded to high expression of Ki67.

Meta-analysis has indicated that high Ki-67 expression is a valuable predictor for advanced TNM stages [9]. This study also showed that there was no difference in PD-L1 expression in pGGO and mGGO lesions and revealed the importance of the GGO component for LUAD. The previous studies stress the importance of GGO absence in the expression of PD-L1, but the expression differences of different GGO were not concluded [13, 35]. X. Yang et al. performed a large study on 2022 nodules from 1844 patients and revealed high PD-L1 expression in 9, 12, and 17% of pGGO, mGGO, and solid nodules; however, they did not compare the difference between pGGO and mGGO; therefore, cannot be compared with the results of the current study [42].

This study indicated that large tumor size and CT texture of pSD in LUAD were correlated with high PD-L1 expression, consistent with the results of previous studies [13–15]. Furthermore, large tumor size and pSD of LUAD also indicated high Ki67 expression, suggesting a relationship between the expressions of Ki67 and PD-L1. Further analysis found that pSD was correlated with the pathological subtype of micropapillary, which was observed both in higher expression of PD-L1 and Ki67. These results were partially consistent with previous reports [29, 43, 44]. However, no association was observed between the expression of PD-L1 and the pathological subtype of solid [23, 45]. In addition, there was no correlation between the expression of Ki67, pSD, and the pathological subtype of solid. These results are controversial and therefore, require further studies.

Interestingly, this study’s result of pGGO and mGGO had similar expression of PD-L1, consistent with studies on the prognosis of LUAD [46–50]. These studies indicated that the GGO predicted favorable OS (overall survival) and DFS (disease-free survival). It has also been revealed that PD-L1 and Ki 67 were correlated with poor prognosis [9, 10, 51]. Furthermore, LUAD with GGO component has been suggested to have low expression of PD-L1 and Ki67, which were poor prognosis biomarkers.

The literature has indicated that PD-L1 could promote cancer growth. Liu S et al. [52] found that the PD-L1 expression in breast cancer tissues correlates with lymph node metastasis. Furthermore, Mu L et al. [53] revealed that tumor-associated fibroblasts promoted the growth of gastric cancer cell lines by upregulating PD-L1 expression. Qu QX et al. [54] revealed that PD-L1 promoted cancer cell growth in ovarian cancer. Moreover, Du W et al. [55] showed that nuclear PD-L1 could promote NSCLC cell proliferation *via* the Growth Arrest-Specific 6 (Gas6)/MerTK signaling pathway. In addition, they also indicated that Nuclear PD-L1 (nPD-L1) coupled with transcription factor Sp1, regulated the synthesis of Gas6 mRNA and promoted Gas6 secretion to activate the MerTK signaling pathway. Activation of MerTK signaling by its ligands Gas6 and Protein S1 (PROS1) promoted cell proliferation.

These aforementioned studies further validate the results of this study. The factors such as smoking, EGFR wild type, and micropapillary promoted the proliferation of LUAD by upregulating the expression of PD-L1, therefore, the expressions of PD-L1 and Ki67 could be significantly related.

The summary of the above data suggests that expression of Ki67 and PD-L1 increased with the progression of cancer, and with the development of cancer, many genes related to Ki67 and PD-L1 are activated and amplified. Furthermore, the expression of Ki67 and PD-L1 act as potent weapons of cancer, which correlated with each other, where the expression of Ki67 promoted cancer, and that of PD-L1 helped tumor cells escape T cell attack. Moreover, Ki67 and PD-L1 become stronger with the progression of cancer. Therefore, the goal should be to reduce these factors to protect patients who have cancer.

### Limitations

(1) It is a retrospective study with a small sample size, which may have caused a selection bias. (2) The cases were limited to LUAD, and other histological subtypes were not addressed. (3) No survival data were provided in the study.

## Conclusion

In summary, this study showed that in LUAD, PD-L1 expression was correlated with male gender, smoking, expression of Ki67, and wild-type EGFR. Furthermore, it was inferred that the high expression of KI67 was the key factor associated with increased PD-L1 and other factors acting *via* KI67.

**Table 1:** Association between clinical characteristics and CT texture with PD-L1 expression

Variable	No. of PD-L1 < 1%	No. of PD-L1 ≥ 1%	*P* value	Univariate OR
Age				
< 60	284	98	…	Reference
≥60	487	176	0.7531	1.05 (0.79–1.40)
Sex				
female	457	131	…	Reference
Male	314	143	0.0011	1.59(1.20–2.10)
Smoking history				
No	724	236	…	Reference
Yes	47	38	< 0.0001	2.48 (1.58–3.90)
Ki67				
Low Expression	484	100	…	Reference
High Expression	287	174	< 0.0001	2.93(2.20–3.91)
EGFR mutation				
Yes	552	159	…	Reference
No	219	115	< 0.0001	1.82(1.37–2.43)
CT texture				
pGGO	262	67	…	Reference
mGGO	248	62	0.91	0.98(0.66–1.44)
pSD	261	145	< 0.0001	2.17 (1.55–3.04)
T stage				
T1a	140	37	...	Reference
T1b	395	130	0.30	1.25(0.82–1.88)
T1c	154	60	0.11	1.47(0.92–2.36)
≥T2	82	47	0.0029	2.17(1.30–3.61)
N stage				
N0	771	272	...	NA
≥N1	0	2	0.97	NA
Pathological Subtypes				
Without lepidic	666	251	...	Reference
With lepidic	104	24	0.040	0.61(0.38–0.98)
Without acinar	100	40	...	Reference
With acinar	670	235	0.52	0.88(0.59–1.30)
Without papillary	642	248	...	Reference
With papillary	128	27	0.007	0.55 (0.35–0.85)
Without micropapillary	615	153	...	Reference
With micropapillary	155	122	< 0.001	3.20(2.40–4.30)
Without solid	641	248	...	Reference
With solid	129	27	0.006	0.54 (0.35–0.84)

Note….data in parentheses are 95% CIs

**Table 2:** Multivariable logistic regression analysis of clinical variables influencing expression of PD-L1

Variable	Odds Ratio		*P* Value
	Point Estimate	95% CI	
Smoking			
No	Reference	NA	…
Yes	1.73	1.04–2.89	0.004
Ki67			
Low	Reference	NA	…
High	2.02	1.44–2.82	< 0.0001
EGFR mutation			
Yes	Reference	NA	…
No	1.52	1.11–2.07	0.009
Micropapillary			
Without			...
With	2.05	1.46–2.89	< 0.0001

Note.…NA = not applicable

**Table 3:** Association between smoking, specific pathological subtypes as well as EGFR status and expression of Ki67

	KI67		*P* Value
	Low expression	High expression	
EGFR Mutation	426	285	...
EGFR Wild	158	176	0.0001
Nonsmokers	549	411	...
Smokers	35	50	0.004
With micropapillary	76	201	< 0.0001
Without micropillary	507	261	...
With solid	90	66	0.60
Without solid	493	396	...

**Table 4:** Association between expression of Ki67 and CT textures as well as Diameter of > 3 cm with ≤ 3 cm in lung adenocarcinoma

	Ki67 High Expression	Ki67 Low Expression	*P* Value
Size			
≤ 3 cm	376	540	…
> 3 cm	85	44	< 0.0001
CT Textures			
pSD	278	128	...
Others	183	456	< 0.0001

Note: …others including pGGO and mGGO.

**Table 5:** Multivariable logistic regression correlation analysis between CT texture of pSD and histopathological subtypes

Variable	Odds Ratio		*P* Value
	Point Estimate	95% CI	
Lepidic			
Without	Reference	NA	…
with	0.30	0.18,0.49	< 0.0001
Acinar			
Without	Reference	NA	…
With	1.11	0.76 1.66	0.58
Mappillary			
Without		NA	...
With	0.51	0.02,11.6	0.67
Micropapillary			
Without	Reference	NA	…
With	3.96	2.52, 5.37	< 0.0001
Solid			
Without		NA	...
With	2.34	0.11,52.0	0.59

## Data Availability

No datasets were generated or analysed during the current study.
